# The Impact of Valsalva Manoeuvres and Exercise on Intracranial Pressure and Cerebrovascular Dynamics in Idiopathic Intracranial Hypertension

**DOI:** 10.1080/01658107.2023.2281433

**Published:** 2023-11-22

**Authors:** Andreas Yiangou, Samuel R. C. Weaver, Mark Thaller, James L. Mitchell, Hannah S. Lyons, Georgios Tsermoulas, Susan P. Mollan, Samuel J. E. Lucas, Alexandra J. Sinclair

**Affiliations:** a Institute of Metabolism and Systems Research, University of Birmingham, Birmingham, UK; b Department of Neurology, University Hospitals Birmingham NHS Foundation Trust, Birmingham, UK; c School of Sport, Exercise and Rehabilitation Sciences, University of Birmingham, Birmingham, UK; d Centre for Human Brain Health, University of Birmingham, Birmingham, UK; e Academic Department of Military Rehabilitation, Defense Medical Rehabilitation Centre, Stanford Hall, UK; f Department of Neurosurgery, University Hospitals Birmingham NHS Foundation Trust, Birmingham, UK; g Neuro-Ophthalmology, University Hospitals Birmingham NHS Foundation Trust, Birmingham, UK

**Keywords:** Cerebrovascular haemodynamics, exercise, idiopathic intracranial hypertension, intracranial pressure, Valsalva maneouvres, cerebrospinal fluid

## Abstract

Idiopathic intracranial hypertension (IIH) is a disease characterised by elevated intracranial pressure (ICP). The impact of straining and exercise on ICP regulation is poorly understood yet clinically relevant to IIH patient care. We sought to investigate the impact of Valsalva manoeuvres (VMs) and exercise on ICP and cerebrovascular haemodynamics in IIH. People with IIH were prospectively enrolled and had an intraparenchymal telemetric ICP sensor inserted. Three participants (age [mean ± standard deviation]: 40.3 ± 13.9 years) underwent continuous real-time ICP monitoring coupled with cerebrovascular haemodynamic assessments during VMs and moderate exercise. Participants had IIH with supine ICP measuring 15.3 ± 8.7 mmHg (20.8 ± 11.8 cm cerebrospinal fluid (CSF)) and sitting ICP measuring −4.2 ± 7.9 mmHg (−5.7 ± 10.7 cmCSF). During phase I of a VM ICP increased by 29.4 ± 13.5 mmHg (40.0 ± 18.4 cmCSF) but returned to baseline within 16 seconds from VM onset. The pattern of ICP changes during the VM phases was associated to that of changes in blood pressure, the middle cerebral artery blood velocity and prefrontal cortex haemodynamics. Exercise led to minimal effects on ICP. In conclusion, VM-induced changes in ICP were coupled to cerebrovascular haemodynamics and showed no sustained impact on ICP. Exercise did not lead to prolonged elevation of ICP. Those with IIH experiencing VMs (for example, during exercise and labour) may be reassured at the brief nature of the changes. Future research must look to corroborate the findings in a larger IIH cohort.

## Introduction

The impact of Valsalva manoeuvres (VMs) and exercise on intracranial pressure (ICP) regulation and physiology in humans is poorly understood. Idiopathic intracranial hypertension (IIH) is a disorder characterised by raised ICP, which drives optic nerve head swelling and a consequent risk of visual loss as well as chronic debilitating headaches.^1,2^ The majority of IIH patients are young females with obesity.^3^ The incidence of IIH is rising, as with the obesity rates (350% increase over the last decade).^4^

Patients with IIH, and their clinicians, are often apprehensive about the impact of VMs and exercise on ICP, and consequently the potential to exacerbate optic nerve swelling and headache. VMs are a function of multiple activities of daily living but, importantly, are also a key aspect to the second stage of labour (this is of particular relevance in a disease which occurs predominantly in women of child bearing age).^5^ The theoretical concern is that VMs during labour could lead to prolonged impact on ICP. There is a historical trend for caesarean section (as opposed to vaginal delivery) in IIH, which is thought to stem from this theoretical concern. Indeed, in the United Kingdom rates of caesarean section have been found to be more than two times higher in women with IIH as compared to the general population.^6^

IIH predominantly occurs in individuals with obesity, and weight loss has been shown to treat IIH by reducing ICP.^7–9^ Therefore, exercise that aims to give additional lifestyle benefits is consequently an important approach in people with IIH. Patients, however, are cautious about exercise due to concerns that it may increase their ICP and consequently exacerbate headaches. This concern is relevant in both non-resistance exercise and in resistance exercise, which commonly induce VMs.^10^ Studies outside of IIH have shown a pattern of changes in cerebrovascular haemodynamics in graded VM including modest brain ischaemia followed by reactive hyperaemia.^11^ The effect of aerobic exercise in ICP is unclear.^12^ Knowledge of the effect of exercise on ICP in IIH would be valuable when discussing the safety of exercise in people with IIH, as there have been no previous studies to guide dialogue. The aim of this study was to evaluate the impact of VMs and aerobic exercise on ICP and cerebrovascular haemodynamics in IIH.

## Materials and methods

### Study design and experimental protocol

This was a sub-study of a prospective clinical study (IIH Pressure Trial: ISRCTN12678718) that was approved by the West Midlands Solihull Research Ethics Committee (17/WM/0179) of which the main findings have been already published.^13,14^ In accordance with the Declaration of Helsinki, all participants gave written informed consent to participate in the study. Participants were recruited from the University Hospitals Birmingham NHS Foundation Trust IIH clinical service.

Women between 18 and 60 years were eligible if they had a diagnosis of IIH^2^ and three participants undertook this sub-study (out of the total fifteen participants of the main study). Numbers were limited due to the study occurring over the COVID pandemic. For this study each participant attended a single-day visit. Participants stopped any ICP-modifying, vasoactive or diuretic drugs with 5.5 drug half-lives prior to the research visit. Participants had not received any surgical intervention for raised ICP, such as a shunt or stent, prior to the research visit. A headache diary was completed 3 days prior and 3 days after the visit, along with headache recordings during the visit. At the study visit, cerebrovascular haemodynamic measures were set up for continuous recording as outlined below. Participants rested in a supine position for 30 minutes to establish a baseline (as had been previously demonstrated for ICP measurements).^14^ Patients then sat up and were given instructions on how to perform a VM in the sitting position. This included instructions to inhale deeply, close mouth, bear down through straining without releasing air (closed glottis),^15^ hold this for 10 seconds, then breath out and resume normal breathing. A minimum of five and a maximum of six VMs were performed by patients with a minimum of 3 minutes rest in between each one. Aerobic exercise was then assessed using a stationary cycle ergometer (Corival, Lode Medical Technology, Groningen, The Netherlands). Participants performed a 30-minute period of moderate intensity exercise at 80% of their estimated maximum heart rate (approximately 65% of maximal aerobic capacity).^16^

### Measurements

As part of the main clinical trial, participants underwent continuous telemetric ICP recording with the Raumedic Intraparenchymal Transdermal Telemetric System (Raumedic, Helmbrechts, Germany).^13^ The telemetric sensor of the system (Neurovent P-tel) was implanted surgically in the right frontal area under general anaesthesia as a day-case procedure. ICP was recorded using the reader (Reader TDT1 readP) with the recordings transmitted and saved to the portable storage unit of the system (Datalogger MPR1), as previously described.^14^ The sampling frequency of this system was at 5 Hz. Measurements were recorded in mmHg and converted into cmCSF (cerebrospinal fluid) by multiplying by 1.36 (the conversion factor).

Participants underwent visual assessments prior to the study visit (visual field testing performed using automated perimetry [Swedish Interactive Threshold Algorithm standard 24–2 strategy, Humphrey Visual Field Analyzer; Carl Zeiss Meditec, Dublin, CA] and optical coherence tomography [OCT] imaging [SPECTRALIS, Heidelberg Engineering, Germany]). Dilated fundus examination was performed by an appropriately trained Neuro-ophthalmology specialist.

Blood velocity of the middle cerebral arteries (MCAv) was measured bilaterally using 2-MHz transcranial Doppler (TCD) ultrasound probes, secured in place using an adjustable headset (DWL, Compumedics Ltd, Germany).

Prefrontal cortical haemodynamics were measured non-invasively using near-infrared spectroscopy (NIRS) (NIRO-200NX, Hamamatsu Photonics KK, Japan). The relative concentrations of oxygenated (O_2_Hb) and deoxygenated haemoglobin (HHb) were obtained along with the tissue oxygenation index (TOI) and total haemoglobin index (THI).

Arterial blood pressure (BP) was measured using finger photoplethysmography (NOVA Finapres Medical Systems, Biomedical Instruments, The Netherlands), and heart rate (HR) using a three-lead electrocardiogram (NOVA). In addition, mean arterial blood pressure (MAP) was estimated using the diastolic and systolic BP via the commonly used formula: MAP = diastolic BP + 1/3(systolic BP – diastolic BP).^17^

All data were acquired continuously via an analogue-to-digital converter (PowerLab ML870; ADInstruments) at 1 kHz. Data were displayed in real-time and recorded for offline analysis using commercially available software (v7.3.8 Lab Chart, ADInstruments). Both positive and negative measurements were recorded. Raw right and left MCAv data were visually inspected to determine signal quality for analysis and if drop-out or noise were seen to be present the most consistent signal was used, otherwise the right MCA was used.

Structured clinical data collected included headache occurrence, characteristics, severity (11-point Numeric rating scale [NRS]: 0–10 where 0 = no pain and 10 = maximum pain; mild = 1–4, moderate = 5–7, and severe = 8–10), and analgesic use. The structured clinical data were recorded during each VM, after rest from the VM, at the beginning of exercise, at 10-minute intervals during exercise and at 30- to 60-minute intervals after exercise during the research visit.

### Data analyses

Baseline data were acquired before each VM for 1 minute and presented as the mean over that minute. The ICP measurements and cerebrovascular haemodynamic assessments were obtained for each of the four VM phases in conjunction with the ICP response (Figures S1 and S2). The phases of a VM are well described in the literature using the blood pressure response, which we have applied here for our analysis of the VM based on the ICP response.^11,18–20^

Specifically, phase I values were calculated as a 2-second average from the start of the VM. Phase II was calculated as the mean over the final 5 seconds of the VM. Phase III was calculated as the mean of the ICP nadir (2 seconds) of the VM. Phase IV was calculated as a 2-second average of the ICP peak following release of the VM.

Baseline recordings represent the mean values over 1 minute before each VM. For the VM phases, the ICP mean represents the mean values of the ICP waveform over 1-second measurements. ICP maximum represents the mean ICP waveform peak values over 1-second measurements. ICP minimum represents the mean ICP waveform trough (nadir) values over 1-second measurements. The ICP Max – Min represents the mean difference between maximum and minimum values, collected as described above, used to represent the waveform amplitude.

The diastolic and systolic phase of the MCAv waveform were analysed along with the mean blood velocity (MCAv_mean_), which was calculated as the mean of the waveform.

An index of cerebral vascular conductance (CVCi) was calculated via the equation MCAv/MAP. The pulsatility index (PI) for MCAv was calculated as (systolic MCAv – diastolic MCAv)/MCAv_mean_.^21^ To calculate change in clinical variables each measurement in different VM phases was compared to the baseline 1-minute recording before the specific VM.

All data are presented as mean ± standard deviation (SD) unless stated otherwise.

## Results

### Baseline characteristics

This prospective clinical study included three participants with IIH, mean age 40.3 ± 13.9 years and IIH disease duration of 8.9 ± 4.2 years (Table 1). The supine and sitting ICP indicated that the IIH was well controlled during the study visits (Table 1). All three participants had headache attributed to IIH with a migraine-like phenotype and tinnitus. At the time of the study visits, no participant reported blurring of vision, visual loss, diplopia or visual obscurations. One participant had active papilloedema (Frisén grade 2), and two participants had a previous diagnosis of IIH (in ocular remission with no papilloedema but other ongoing symptoms including headache) prior to the study visit.

**Table 1.** Baseline characteristics.

Clinical parameter	Mean (SD), *n* = 3	
Age at visit (years)	40.3 (13.9)	
BMI (kg/m^2^)	40.9 (3.8)	
Perimetric mean deviation (dB)	0.40 (0.31)	
OCT global peripapillary RNFL thickness (µm)	104.3 (26.7)	
IIH disease duration (years)	8.9 (4.2)	
	mmHg	cmCSF
ICP (supine)	15.3 (8.7)	20.8 (11.8)
ICP (sitting)	−4.2 (7.9)	−5.7 (10.7)
Maximum ICP (sitting)	7.1 (8.8)	9.7 (12.0)
Minimum ICP (sitting)	−11.8 (7.6)	−16.0 (10.3)
Maximum – minimum ICP (sitting)	19.0 (4.1)	25.8 (5.6)

BMI = body mass index; CSF = cerebrospinal fluid; ICP = intracranial pressure; IIH = idiopathic intracranial hypertension; OCT = optical coherence tomography = RNFL: retinal nerve fibre layer thickness; SD = standard deviation.

### Valsalva manoeuvre

**Figure 1.**
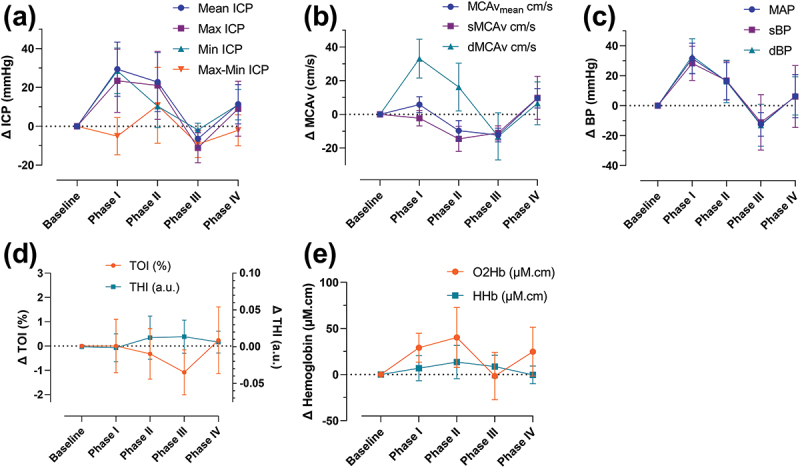
Change in cerebrovascular haemodynamics during different phases of the Valsalva manoeuvre compared with baseline. (a) Changes in intracranial pressure. (b) Changes in middle cerebral artery blood velocity. (c) Changes in blood pressure. (d) Changes in tissue oxygenation index and total haemoglobin index. (e) Changes in oxygenated haemoglobin and deoxygenated haemoglobin.

There was a substantial increase in the mean ICP in phase I of the VM, increasing from baseline (sitting) by 29.4 ± 13.5 mmHg (40.0 ± 18.4 cmCSF) (Figure 1a, Table 2), which remained elevated through phase II (increased 22.7 ± 14.8 mmHg from baseline). Mean ICP reduced rapidly upon release of the VM (phase III), with mean ICP −6.5 ± 4.5 mmHg below baseline measures. At phase IV, the mean ICP increased by 11.3 ± 9.8 mmHg above baseline. This pattern was also seen in the maximum and minimum ICP outcomes (Figure 1a, Table 2). In addition, the amplitude (ICP Max – Min) of the waveform also changed. There was an ICP amplitude reduction at phase I, an increase at phase II and reductions in phases III and VI (Figure 1a, Table 2).

dBP = diastolic blood pressure; dMCAv = diastolic middle cerebral artery blood velocity; HHb = deoxygenated haemoglobin; ICP = intracranial pressure; MAP = mean arterial blood pressure; MCAv = middle cerebral artery blood velocity; O2Hb = oxygenated haemoglobin; sBP: systolic blood pressure; sMCAv = systolic middle cerebral artery blood velocity; THI = total haemoglobin index; TOI = tissue oxygenation index.

**Table 2.** Clinical variables at baseline and at different phases of the Valsalva manoeuvre.

Clinical variable – mean (SD) (*n* = 17, unless otherwise specified)*	Baseline	Phase I	Phase II	Phase III	Phase IV
ICP mean sitting-up (mmHg)	−4.2 (7.9)	25.1 (19.8)	18.5 (21.5)	−10.7 (8.9)	7.1 (13.4)
ICP maximum (mmHg)	7.1 (8.8)	30.5 (21.0)	28.2 (22.3)	−3.9 (11.6)	16.1 (18.3)
ICP minimum (mmHg)	−11.8 (7.6)	16.7 (17.2)	−1.5 (16.3)	−13.7 (8.2)	−0.8 (10.2)
ICP max – min (mmHg)	19.0 (4.1)	13.8 (7.2)	29.7 (17.7)	9.8 (5.1)	16.9 (8.8)
MCAv_mean_ (cm/s) (*n* = 10)	55.9 (7.6)	61.7 (10.3)	46.2 (5.7)	43.6 (5.1)	65.5 (12.3)
Systolic MCAv (cm/s) (*n* = 10)	83.7 (9.8)	83.5 (13.3)	69.8 (7.6)	74.4 (12.7)	96.0 (21.6)
Diastolic MCAv (cm/s) (*n* = 10)	42.3 (6.0)	47.3 (8.7)	35.9 (4.8)	32.1 (4.0)	48.9 (7.9)
Pulsatility index (*n* = 10)	0.8 (0.1)	0.6 (0.1)	0.7 (0.1)	1.0 (0.2)	0.7 (0.1)
CVCi (*n* = 8)	0.47 (0.05)	0.41 (0.04)	0.36 (0.04)	0.43 (0.07)	0.47 (0.08)
MAP (mmHg) (*n* = 14)	109.6 (5.6)	141.1 (9.2)	126.0 (10.8)	97.2 (6.8)	116.0 (16.4)
Systolic BP (mmHg) (*n* = 14)	157.6 (12.9)	185.9 (19.1)	174.4 (20.9)	146.4 (14.1)	163.6 (10.3)
Diastolic BP (mmHg) (*n* = 14)	85.6 (11.2)	118.8 (12.6)	101.9 (14.6)	72.6 (10.1)	92.2 (20.6)
Heart rate HR (beats/min) (*n* = 14)	70 (3)	79 (7)	81 (11)	83 (5)	69 (4)

*n represents number of VMs.

Phase I values were calculated as a 2-second average from the start of the VM. Phase II was calculated as the mean over the final 5 seconds of the VM. Phase III was calculated as the mean of the ICP nadir (2 seconds) of the VM. Phase IV was calculated as a 2-second average of the ICP peak following release of the VM. Baseline recordings represent the mean values over 1 minute before each VM. For the VM phases the ICP mean represents the mean values of the ICP waveform over 1-second measurements. ICP maximum represents the mean ICP waveform peak values over 1-second measurements. ICP minimum represents the mean ICP waveform trough (nadir) values over 1-second measurements. The ICP max – min represents the mean difference between the peak and trough (nadir) values over 1-second measurements.

BP = blood pressure; CVCi = cerebrovascular conductance index; ICP = intracranial pressure; MAP = mean arterial blood pressure; MCAv = middle cerebral artery blood velocity; VM = Valsalva manoeuvre.

The changes in mean ICP were coupled with the changes in BP, evidenced by the pattern of changes through the VM phases (Figure 1c, Table 2).

The similar pattern of change with the ICP and BP was noted predominantly in diastolic MCAv, and to a lesser extend in MCAv_mean_ and systolic MCAv, where a reduction was noted in phase II of the VM (Figure 1b). The HR demonstrated an increase compared with baseline through phases I, II and III of the VM and was similar to baseline at phase IV (Table 2).

NIRS measures of prefrontal TOI showed a decrease through phases II and III, returning close to baseline at phase IV, whilst for THI an increase was seen in phases II and III (Figure 1d). A pattern of changes similar to the ICP was seen for O_2_Hb during the phases of the VM (Figure 1e). The HHb increased in the VM phases I, II and III (Figure 1e).

**Figure 2.**
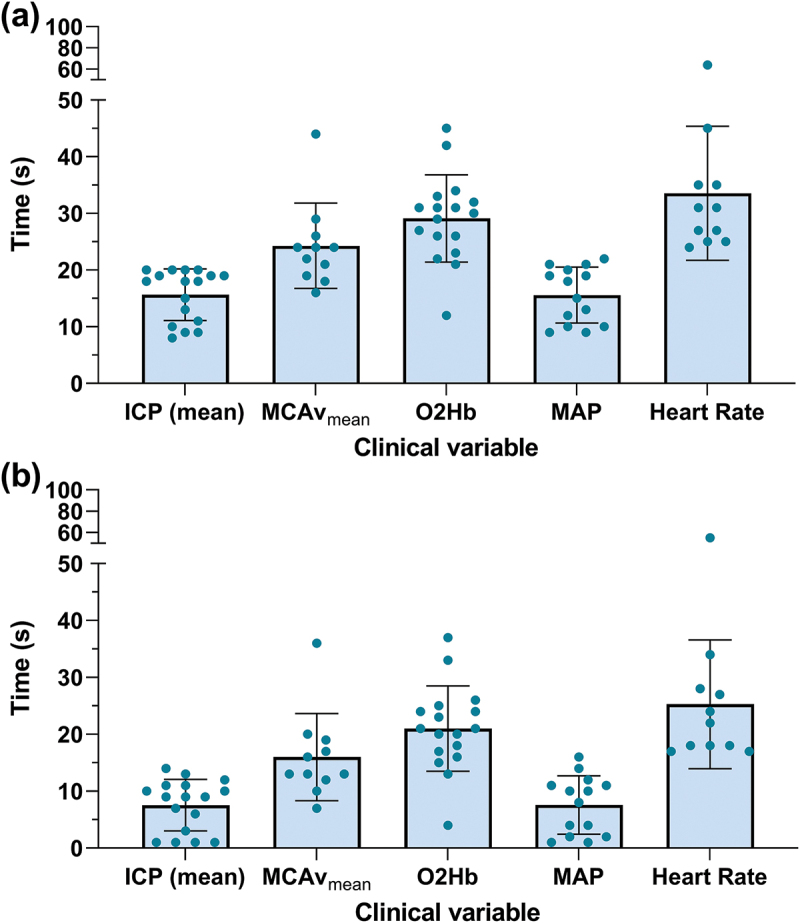
Time of clinical variables to return to baseline after Valsalva manoeuvres. (a) Time of clinical variables to return to baseline from the beginning of the Valsalva manoeuvres (Phase I). (b) Time of clinical variables to return to baseline from the release of Valsalva manoeuvres (Phase III).

The mean time for the mean ICP to return to baseline after VMs in our cohort was 15.6 ± 4.3 seconds, which was similar to MAP (Figure 2a). The mean MCAv_mean_ and O_2_Hb took 24.2 ± 6.9 seconds and 29.1 ± 7.2 seconds, respectively, to return to baseline after VMs (Figure 2a). The mean time of the ICP to return to baseline from the beginning of phase III was 7.6 ± 4.3 seconds (Figure 2b).

ICP = intracranial pressure; MAP = mean arterial blood pressure; MCAv = middle cerebral artery blood velocity; O2Hb = oxygenated haemoglobin.

### Exercise

During exercise, HR rose by 78 ± 9 beats/minute confirming that participants had successfully achieved 80% of their age-predicted maximal HR. Across all three participants, this intensity was reached within 7 minutes.

**Figure 3.**
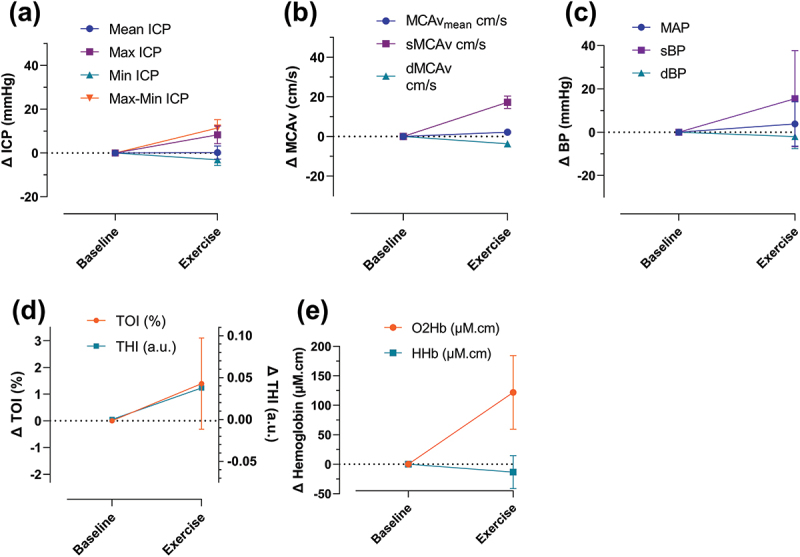
Change in cerebrovascular haemodynamics during exercise compared with baseline. (a) Changes in intracranial pressure. (b) Changes in middle cerebral artery blood velocity (c) Changes in blood pressure. (d) Changes in tissue oxygenation index and total haemoglobin index. (e) Changes in oxygenated haemoglobin and deoxygenated haemoglobin.

The change in mean ICP was not meaningful at 0.2 ± 2.4 mmHg; however, an increase was seen in the maximum ICP (8.3 ± 3.3 mmHg), alongside a decrease in the minimum ICP of −3.1 ± 2.1 mmHg (Figure 3a). This resulted in an increase in the amplitude of the waveform during the exercise of 11.4 ± 3.2 mmHg (Figure 3a). As with the increase in the maximum ICP, there was an increase in the systolic MCAv and systolic BP compared with baseline of 17.3 ± 2.2 cm/s and 15.4 ± 18.2 mmHg, respectively (Figure 3b,c). The diastolic MCAv and diastolic BP were slightly reduced during exercise by −3.7 ± 0.3 cm/s and −2.0 ± 4.6 mmHg, respectively, whilst MCAv_mean_ and MAP did not substantially change during exercise (Figure 3b,c).

dBP = diastolic blood pressure; dMCAv = diastolic middle cerebral artery blood velocity; HHb = deoxygenated haemoglobin; ICP = intracranial pressure; MAP = mean arterial blood pressure; MCAv = middle cerebral artery blood velocity; O2Hb = oxygenated haemoglobin; sBP: systolic blood pressure; sMCAv = systolic middle cerebral artery blood velocity; THI = total haemoglobin index; TOI = tissue oxygenation index.

NIRS measures of cerebral haemodynamics demonstrated an increase in TOI (1.4 ± 1.4%), THI (0.04 ± 0.00 a.u.) and O_2_Hb (121.9 ± 51.1 μM·cm), whilst HHb was slightly reduced (−13.3 ± 22.9 μM·cm; Figure 3d,e).

### Headache

Only one participant reported headaches immediately prior to the study visit (over the preceding 3 days), which were of a mild-to-moderate severity and did not require any analgesic relief. The headaches did not worsen after the visit. This participant was the only one who reported mild self-limiting head pain at 1 minute after the VMs that was both pulsing and pressure-like in nature. Similar headache was noted at the start of exercise with no associated features. These headache reports were not different from the headaches reported prior to the study visit. No headache was reported by the other two participants prior, during or after the research study visits.

## Discussion

This study has provided a unique opportunity to investigate the coupled relationship between ICP and cerebrovascular dynamics during VM and exercise in IIH. We observed a substantial rise in the mean ICP during the VM with a mean rise of 29 mmHg (40 cmCSF) to the peak of the VM, rapidly returning to baseline within 16 seconds. The pattern of changes in the ICP during the VM was coupled with the changes in BP and, in particular, diastolic MCAv. Exercise had little effect on the mean ICP values.

The characterisation of the cerebrovascular changes during VMs in this cohort is of particular relevance as VMs are part of activities of daily living, such as coughing, sneezing and defaecation. A brief rise of 40 cmCSF is substantial, however the return to baseline within 16 seconds from initiation is reassuring for people with IIH. Specifically, as IIH mostly affects women of childbearing age, the swift return to baseline of the ICP is of key relevance for the second stage of labour that often includes a similar VM of comparable duration.^5,22,23^ Based on the recent recommendations of three to four pushing efforts of 6 to 8 seconds per contraction (over 60 to 90 seconds), our data indicate that ICP will likely return to baseline between the pushing efforts in the second stage of labour.^22,23^ Therefore, these data from this small sample size provide support for the current existing IIH guidance.^2,24^ These recommend that method of delivery should not be based solely on a previous diagnosis of IIH and perceived risk of VMs on IIH for the majority of patients.^2,24^

There is strong evidence to suggest that IIH is a condition that is best managed by weight loss and negative energy expenditure,^7–9^ therefore advice on lifestyle modifications and exercise for people with IIH is common in the clinical setting.^2^ Our data are reassuring as no increase in mean ICP was noted during moderate aerobic exercise. This supports the rational for IIH patients being able to exercise without a lasting impact on their ICP and therefore providing the opportunity to access the benefits of regular exercise.

Headache burden in IIH patients drives morbidity.^25^ Headaches can be driven by VM or exercise (The International Classification of Headache Disorders, third edition, primary cough headache and primary exercise headache).^26^ We hypothesised that elevations of ICP during VM or exercise could cause exacerbation of headache. There were too few patients in this study to evaluate this aspect. However, it is reassuring that ICP changes were so short-lived in VM and clinically minimal during exercise and hence we suggest that it would be unlikely that VMs or exercise meaningfully impact long-term headache severity.

ICP changes during VMs were closely coupled with the changes in BP, which suggests the potential utilisation of BP measurements (using finger plethysmography or alternate continuous BP measures) as a proxy for direct measurement of ICP, during VMs. Of note, the changes of mean ICP and MAP during aerobic exercise were not substantial, but a similar trend was noted in the changes of maximum ICP with the measures of systolic BP and systolic MCAv. Future studies should seek to validate this approach, to enable non-invasive BP monitoring to be used as an initial bedside assessment tool to screen for potential variability in ICP prior to direct measurement in the context of a VM or other activities. Indeed, studies have previously supported the use of cerebrovascular assessments to predict ICP.^27,28^

The changes in cerebrovascular haemodynamics during the VM are in line with existing literature for the healthy population.^11^ This includes the pattern of changes seen in diastolic MCAv during the VM which was analogous to the changes in ICP. This indicates that the diastolic component of the MCAv may be the predominant driver of the rise in ICP. A negative correlation between ICP and diastolic MCA velocity was noted in a larger study of patients with brain injury undergoing surgery.^28^ The different results may represent differences in brain pathology which subsequently would affect cerebral perfusion pressure, cerebrovascular resistance, compliance and hence cerebral blood flow auto-regulation.^28^ The pattern of ICP changes during the different VM phases in our study was further akin with the changes in MAP, sBP, dBP and O_2_Hb (Figure 1). A similar pattern was seen with the increase in BP and MCAv with Perry et al.^11^ at the onset of the VM; however, for the subsequent phases our results are of different magnitude. The difference in magnitude of the changes between cerebrovascular haemodynamic assessment also resulted in differences in the pattern of changes during the VM in calculated indices of CVCi and PI.^11^ This may represent differences in the population (such as age, disease) or variability of the VM in our study versus that of Perry et al., who used a pressure transducer to help guide VM-induced pressure changes.^11^ These technical factors are likely of importance as the level and duration of the strain, the body position, breathing pattern can affect the cardiovascular and hence the cerebrovascular response to the VM.^29^

Consistent with our observations, a similar increase in ICP of 31 mmHg (42 cmCSF) was noted in a study of seven participants (varying intracranial pathologies) from ventricular catheter ICP monitors during a VM; however, no rise in BP was reported.^10^ In another study, an estimated rise in ICP of 21 mmHg (28 cmCSF) during modified VM (supine) was documented in six paediatric patients (varying intracranial pathologies) with a sensor reservoir ICP monitor.^30^ Further, there was a rise in the waveform amplitude of an estimated 6 to 7 mmHg (8 to 9 cmCSF), whilst in our study a substantial fluctuation of amplitude was noted between the phases (range between 9.8 and 29.7 mmHg; see Table 2); however, the difference between these studies may represent a difference of waveform measurements and technical factors of the VM described above.^29,30^ The amplitude changes seen in VM resulted from disproportionate changes between the maximum and minimum ICP compared with baseline. Finally, the rise in ICP during the VM we observed is also corroborated by those of Neville and Ergan,^31^ who used manometry measurements by lumbar puncture in a study of 15 healthy individuals and reported that ICP increased by an estimated mean 13 mmHg (18 cmCSF) during VMs.^31^ In our study we were able to demonstrate an increase in ICP during VMs and further demonstrated in detail the changes during each of the VM stages, which has not been performed before in anyone with a CSF disorder. The mean increase of 40cmCSF in ICP during VMs further emphasises the importance of ensuring that the patient is in the correct left lateral decubitus position breathing normally with relaxed abdomen during diagnostic lumbar puncture to avoid misdiagnosis.^31^

Two studies, with 8 and 20 patients, respectively, evaluated ICP and exercise in people with varying intracranial pathologies and demonstrated no substantial increase in ICP (measured with a ventricular or parenchymal catheter) during low-intensity exercise in an intensive care setting.^12,32^ Nevertheless, the results of these studies are difficult to compare to our study as the exercise intensity was much lower (HR did not increase) and the ‘exercise’ used in this clinical setting is not comparable with usual daily exercise activities.^12,32^ A web-based survey performed in IIH patients (*n* = 218) in 2016 reported that a high proportion of IIH patients have barriers to exercise, mainly due to symptom exacerbation and pain.^33^ Whilst we did not observe a substantial increase in ICP during exercise, the wider waveform noted and changes in blood flow may be of clinical relevance and should guide further investigations. Aerobic exercise does not have the same effects on cerebrovascular physiology as a VM or resistance exercise, also noted in a healthy volunteer study over 3 months.^34^ VM or resistance exercise have been demonstrated to have different effects on brain perfusion including changes in cerebrovascular resistance, which may account for the differences in changes in ICP.^34^ In our study, the rise in systolic MCAv and systolic BP was similar to the rise of the maximum ICP during exercise, whereas in the VM the changes in diastolic MCAv may be the driver for the changes in ICP.

There are limitations to our study, which must be considered. The observations of this study were derived from only three patients with well-controlled IIH over a mean disease duration of 9 years and body mass index of 40.9 kg/m^2^. Therefore, results cannot be generalised to other IIH disease stages or patient cohorts, e.g. cohorts with much higher ICP or acute IIH phase at diagnosis or at remission after weight loss. These patients would warrant further evaluation. Participant recruitment was limited by the reduced participants due to the COVID-19 pandemic along with ICP monitor functionality and availability. Therefore, future research must look to corroborate the findings in a larger IIH cohort including patients with acute and chronic disease and varying disease activity. Due to the reduced participants, the interpretation of headache features is particularly limited. It would also be of interest to compare the full spectrum of the disease including those with higher ICPs to those with well-controlled IIH. A larger study would allow more extensive analysis with increased statistical rigour, alongside exploration of how variations in pathology modify the responses seen. Our study, however, was unique as it provided a continuously recorded ICP alongside cerebrovascular haemodynamic assessments in real-time in patients with IIH. We further noted that the intensity of VM can vary between individuals and between the VM of each individual, hence this could have increased the variability of our results. However, the pattern of cerebrovascular haemodynamic changes through the stages of the VM were similar to other published reports of standardised VM and real-time recording of physiology measures allowed monitoring of the participants’ effort during the VM.^11^

In conclusion, this is the first report to investigate the effects of VMs and exercise in IIH. A substantial increase of ICP during a VM was noted with a similar pattern of changes to the BP and MCAv. Importantly, there was no lasting effect on ICP after a VM, as the increase was short-lived. The lack of change in mean ICP during exercise is of relevance to patients who are often concerned that aerobic exercise could elevate their ICP. The results are pertinent to patient care, as the data provide reassurance that exercise and VMs have only transient impact on ICP. This is also reassuring in the context of understanding changes in ICP that might occur during labour in IIH.

## Supplementary Material

Supplemental Material

## Data Availability

Anonymised individual participant data can be made available along with the main trial protocol and main trial statistical analysis plan. Proposals should be made to the corresponding author and will be reviewed by the Data Sharing Committee in discussion with the Chief Investigator. A formal Data Sharing Agreement may be required between respective organisations once release of the data is approved and before data can be released.
