# Supplementary material for: The Impact of Valsalva Manoeuvres and Exercise on Intracranial Pressure and Cerebrovascular Dynamics in Idiopathic Intracranial Hypertension
Source: Neuroophthalmology. 2023 Nov 22;48(2):122–33. doi: 10.1080/01658107.2023.2281433 (PMC10936629; doi:10.1080/01658107.2023.2281433)
Supplement: Supplemental Material [file IOPH_A_2281433_SM4694.zip › Supplemental_Figure_legends.docx]

**Supplemental Figure 1.** Sample graphic of the ICP trace during a Valsalva maneuver (VM) and the phases.

Phase I values were calculated as a 2-second average from the start of the VM. Phase II was calculated as the mean over the final 5 seconds of the VM. Phase III was calculated as the mean of the ICP nadir (2 seconds) of the VM. Phase IV was calculated as a 2-second average of the ICP peak following release of the VM. The rectangular border around phases I and II represents the onset of the VM until up to the release.

ICP: Intracranial pressure; BL: Baseline

**Supplemental Figure 2.**

Raw sample trace showing the changes in intracranial pressure (ICP) and cerebrovascular haemodynamics during a Valsalva maneuver (VM).

Initiation of a VM is denoted by the number 1 dashed vertical line and the end of the VM denoted by the number 2 dashed vertical line.

ICP: intracranial pressure; s/dMCAv: systolic/diastolic middle cerebral artery blood velocity; BP: systolic/diastolic blood pressure; TOI: tissue oxygenation index; THI: total haemoglobin index; O2Hb: Oxygenated haemoglobin; HHb: Deoxygenated haemoglobin; HR: heart rate
