# Supplementary figures and images for: The Impact of Valsalva Manoeuvres and Exercise on Intracranial Pressure and Cerebrovascular Dynamics in Idiopathic Intracranial Hypertension
Source: Neuroophthalmology. 2023 Nov 22;48(2):122–33. doi: 10.1080/01658107.2023.2281433 (PMC10936629; doi:10.1080/01658107.2023.2281433)

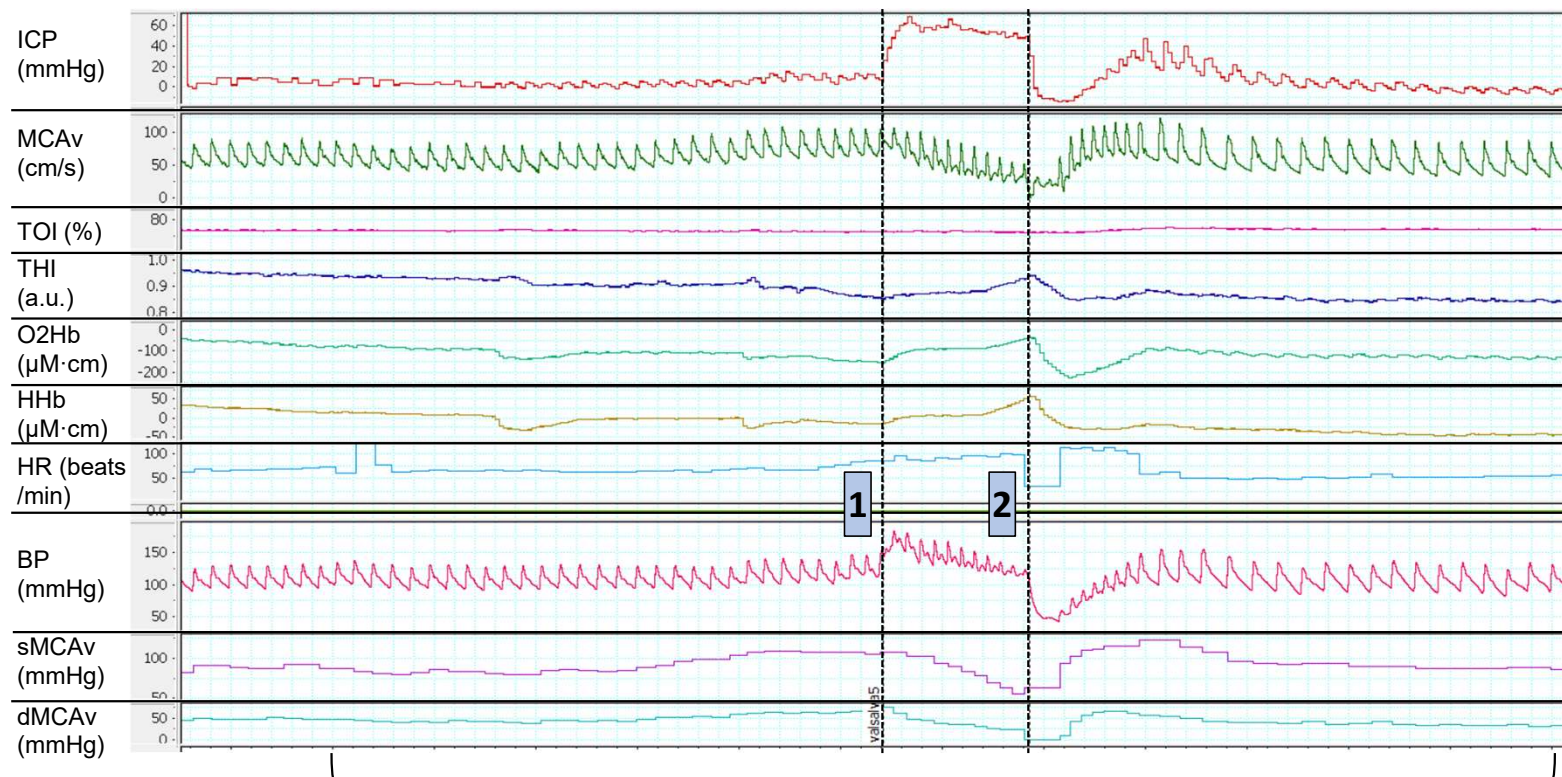

Time (60 seconds)

Supplement: Supplemental Material [file IOPH_A_2281433_SM4694.zip › Suppl._Figure_2_V0.2.pdf]

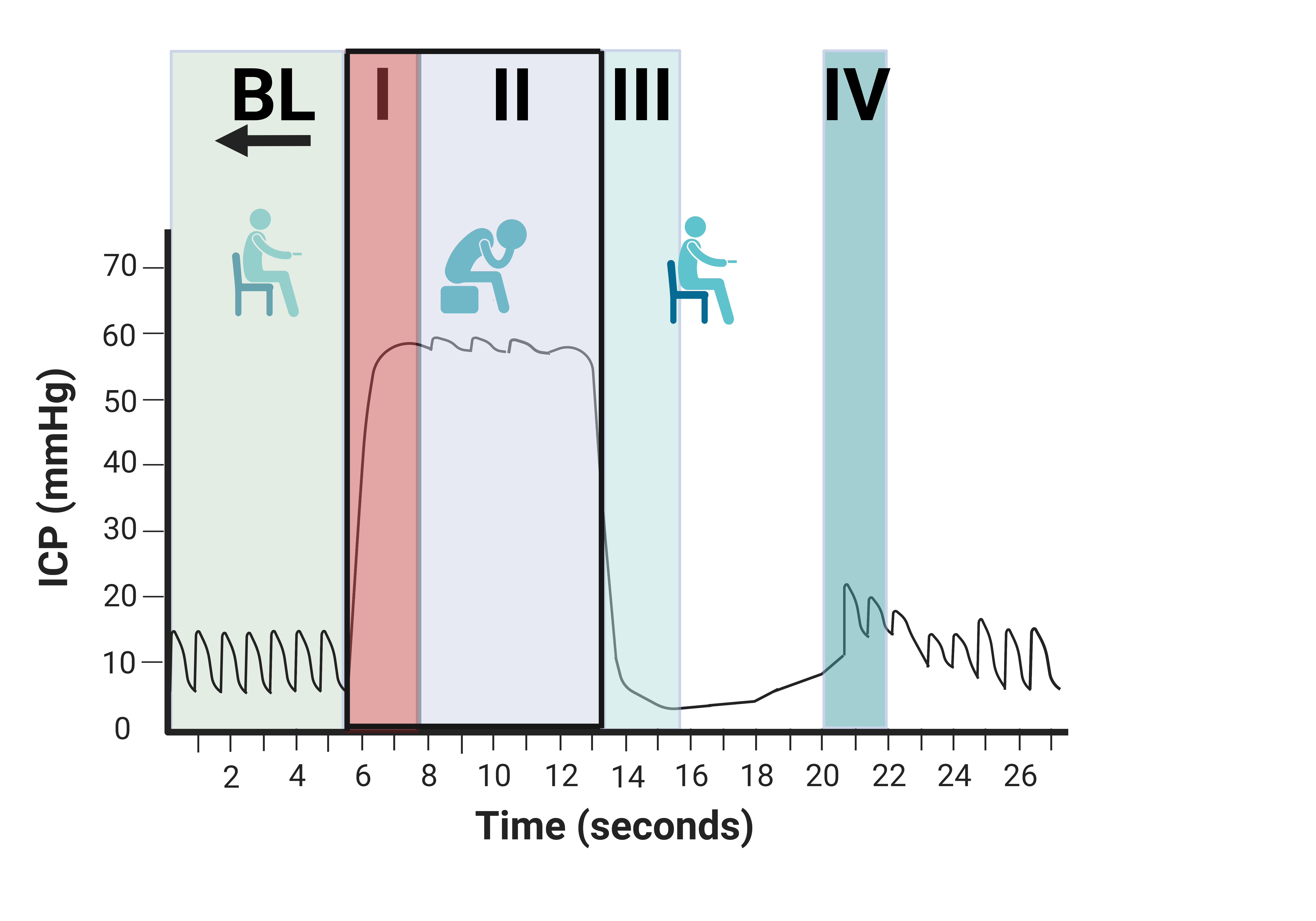

Supplement: Supplemental Material [file IOPH_A_2281433_SM4694.zip › Supple._Figure_1_BioR.jpg]
